# The impact of epidemics on inland development in Qing Taiwan, 1684-1895

**DOI:** 10.1590/S0104-59702025000100048

**Published:** 2025-10-20

**Authors:** Junling Huang, Qiying Wang

**Affiliations:** i Associate professor, Department of History/Xiamen University. Xiamen City – Fujian Province – China; ii Assistant professor, Department of History/Xiamen University. Xiamen City – Fujian Province – China. qiyingw@gmail.com

**Keywords:** Qing dynasty, Taiwan, Epidemics, Inland development, Dinastia Qing, Taiwan, Epidemias, Desenvolvimento do interior

## Abstract

During the early Qing dynasty, Taiwan remained largely undeveloped, with vast areas covered in dense wilderness and forests. The terms *zhangli* and *zhangyi*, which appear frequently in historical documents, most likely cover a variety of epidemic diseases such as malaria, dysentery, cholera, and scarlet fever. Following the Opium Wars, as Western powers increased pressure on Taiwan, the Qing government sought to intensify inland development. However, persistent epidemics in the mountainous regions hindered efforts to “open the mountains and pacify the aborigines.” These health challenges significantly affected Taiwan’s development during the Qing dynasty, experiences which share many similarities with the history of South America.

## Resumo

Durante o início da dinastia Qing, Taiwan permaneceu amplamente subdesenvolvido, com vastas áreas cobertas por densas florestas e regiões selvagens. Os termos *zhangli* e *zhangyi*, frequentemente encontrados em documentos históricos, provavelmente abrangem uma variedade de doenças epidêmicas, como malária, disenteria, cólera e escarlatina. Após as Guerras do Ópio, à medida que as potências ocidentais aumentavam a pressão sobre Taiwan, o governo Qing buscou intensificar o desenvolvimento do interior. No entanto, epidemias persistentes nas regiões montanhosas dificultaram os esforços para “abrir as montanhas e pacificar os indígenas”. Esses desafios sanitários afetaram significativamente o desenvolvimento de Taiwan durante a dinastia Qing, experiências que compartilham muitas semelhanças com a história da América do Sul.

The scholarly literature examining the interrelationship between migration, environmental change and disease remains relatively underdeveloped. Since the Age of Discovery, the impact of population displacement and migration (particularly within the context of environmental transformations) on the prevalence and spread of disease has been recognized, ultimately catalyzing the emergence of tropical medicine as a modern scientific response. These dynamics have manifested across both the Western and Eastern hemispheres.

Taiwan, situated on the southeastern periphery of China, experienced relatively late development. In the 21st year of Emperor Kangxi’s reign (1682), General Shi Lang led the Qing naval forces in defeating the remnants of the Ming loyalist regime under Zheng Keshuang, thereby consolidating Qing authority over the island and completing the dynastic transition from the Ming to the Qing. At this time, Taiwan’s population remained sparse; according to rough estimates by the Dutch East India Company in 1652, the central and western regions of Taiwan contained approximately 251 indigenous villages with a widely dispersed total indigenous population of 59,805 (Jiang, 2003, p.284). The Han immigrant population was estimated at fewer than fifty thousand. The island, spanning 36,000 square kilometers, was largely uncultivated and undeveloped. In 1683, the Qing government formally incorporated Taiwan into its administrative system, establishing one prefecture and three counties. By the time Taiwan was designated a province in 1885, the island’s population had surged to nearly two million. However, throughout this period epidemic diseases posed a significant obstacle to Han settlers from Fujian and Guangdong, who played a central role in the island’s cultivation and development.

The environmental challenges of Taiwan, particularly its tropical climate and dense forests, created a “miasmatic” environment that was highly conducive to the spread of diseases such as malaria, dysentery, and cholera. This environmental determinism shaped the island’s epidemiological history, as the Qing dynasty struggled to manage the health risks posed by Taiwan’s undeveloped interior. Prior to the advent of modern medicine, the classification and nomenclature of epidemics in China were often inconsistent and imprecise, despite the severity of their impact. In traditional Chinese society, epidemic outbreaks – frequently referred to as plagues – were regarded as a form of “natural disaster.” Unlike earthquakes, storms, or volcanic eruptions, epidemics distinguished themselves through their rapid spread and their ability to inflict prolonged suffering. Their geographical reach often exceeded that of other natural calamities. Recent scholarship has increasingly explored the intersections between colonialism, modern medicine, epidemic outbreaks, and regional cultural transformations. In particular, historians have examined the role of medicine in the expansion of Western colonial empires, the transmission of Western medical practices to Africa, Asia, Latin America and the Pacific via colonial administration, missionary activity, and trade networks, as well as the reciprocal influence of local disease environments on colonial governance. Furthermore, scholars have investigated the ways in which Western medicine reshaped the social structures, cultural practices and economic systems of colonized societies, often in complex dialogue with indigenous medical traditions.

Robert Peckham’s *Epidemics in Modern Asia* (2016) traces the history of epidemics in Asia from the nineteenth century to the present, analyzing their profound social, political, and cultural ramifications. The volume interrogates the interplay between health crises and colonialism, nation-building, globalization, and public health governance. *Disease, medicine and empire*, edited by Roy MacLeod and Milton Lewis (2022), investigates the relationship between disease, medical practices, and the expansion of European empires from the eighteenth to the twentieth centuries, illustrating how public health strategies were shaped by imperial imperatives and, in turn, influenced colonial societies. John McNeill’s *Mosquito Empires: ecology and war in the Greater Caribbean, 1620-1914* (2010) offers an ecological perspective on the geopolitical history of conquest, imperial rivalry, and revolution in the Greater Caribbean. This work presents a compelling paradigm for understanding how endemic pathogens shaped the course of international history and represents a significant contribution to the historiography of disease and empire in the Americas. Despite the richness of this scholarship, English-language studies that examine similar dynamics within the context of China remain comparatively scarce. While Chinese scholars have made significant strides in investigating the relationships between ecological transformations, epidemic outbreaks, and geopolitical developments within China, notable gaps persist in the literature. The case of Taiwan during the Qing dynasty, with its distinct historical and ecological conditions, warrants further exploration.

The Qing dynasty’s inability to effectively manage Taiwan’s disease environment highlights the limitations of traditional Chinese medicine in the face of tropical diseases. This failure underscores the broader theme of ecological imperialism, where environmental factors played a decisive role in shaping colonial and imperial outcomes. This article employs the concept of ecological imperialism to move beyond the simplistic, linear explanations of traditional environmental determinism (such as Jared Diamond’s theory of geographical determinism). Instead, it emphasizes the dynamic interplay between colonizers and the colonized environment: namely that imperial expansion was not only constrained by environmental conditions but also sought to reshape ecological orders through tools such as medical knowledge and land transformation (drawing on Alfred Crosby’s original definition of ecological imperialism). In the case of Taiwan, this bidirectional interaction is evident; the Qing dynasty’s rule was limited by the malarial and pestilential environment, yet it also attempted to reconstruct the ecological frontier through policies such as *Kaishan Fufan/*开山抚番 [“Opening the mountains and pacifying the Indigenous people”]. During the early to mid-Qing dynasty, the government adopted a policy of restricting development in the central and eastern mountainous regions of Taiwan. This was primarily due to concerns that Han Chinese migrants would encroach upon indigenous lands, leading to conflicts between the two groups. However, by the late nineteenth century, the vast, undeveloped mountain forests of Taiwan had become administrative voids that provided foreign powers such as Japan a pretext to invade, claiming that these areas were “ownerless” and unoccupied. In response, the Qing government implemented the *Kaishan Fufan* policy to strengthen development and administrative control over the mountainous interior. Faced with resistance from indigenous communities, the Qing authorities mobilized military forces and civilian settlers to “civilize” and manage the mountain tribes. However, the harsh natural environment, prevalence of epidemics, resistance from indigenous groups, and limited effectiveness of traditional Chinese medicine in disease prevention and treatment all posed serious challenges; these factors constrained the Qing government’s efforts to implement its new policy and left large portions of the mountainous interior beyond effective state control.

Existing scholarship on epidemics in Qing-era Taiwan remains relatively limited. The studies in the literature predominantly focus on two aspects: identifying the underlying causes of epidemic outbreaks and analyzing the strategies employed by Taiwanese society to mitigate their impact. While this research provides foundational insights into the public health challenges of the period, the topic remains fertile ground for further inquiry (Lin, Lin, 2014). Several studies also examine natural disasters in Fujian and Taiwan during the Qing dynasty, often situating epidemic outbreaks within broader environmental contexts. These works highlight the interplay between disease outbreaks and other ecological phenomena, contributing to a more comprehensive understanding of how epidemics shaped societal responses and public health policies (Xu, 2004, 2005). While much of the existing research offers a broad overview of epidemic outbreaks and responses in Taiwan during the Qing period, this study focuses on the late nineteenth century, a critical juncture when the Qing Empire responded to growing pressure from Western colonial powers by attempting to accelerate the development of Taiwan’s interior. But despite the Qing court’s ambitions to exert greater control over the island’s mountainous regions, it lacked the capacity to effectively manage the concurrent spread of epidemic diseases.

In the early and mid-Qing period, the government prioritized stability in Taiwan, restricting Han migration into the interior to prevent conflict with indigenous communities. However, the Mudan incident of 1874 underscored the geopolitical vulnerability of Taiwan’s interior and prompted the Qing authorities to intensify their efforts to develop the region, much as they had strengthened coastal fortifications and expanded naval power. Yet indigenous communities in the interior retained cultural practices such as nudity, tattooing, headhunting, and consumption of raw food. While the Qing government relied on military forces to construct roads and maintain security against indigenous resistance, epidemic outbreaks frequently disrupted these efforts, significantly impeding the Qing court’s *Kaishan Fufan* initiatives. Lacking modern medical knowledge and institutional frameworks, the Qing administration was constrained to traditional Chinese medicine, which proved inadequate in addressing the epidemiological challenges posed by the island’s environment. The Qing Empire’s broader institutional, economic, and military limitations ultimately rendered it incapable of fully realizing its ambitions for Taiwan’s interior development or fortifying the island against foreign encroachment. After its 1895 defeat in the First Sino-Japanese War, the Qing court was compelled to cede Taiwan to Japan, marking the island’s transition into a colonial possession. This history reveals the complex relationship between governmental decision-making, regional development, epidemics, and medical responses in nineteenth-century East Asia, particularly in Taiwan’s response to colonialism.

## Epidemics in Qing Taiwan: frequent outbreaks and continuous threats

Even before the Qing period, epidemics occasionally occurred in Taiwan. Since the indigenous tribes of Taiwan did not have a written language, the earliest primary documents related to Taiwan’s history began to appear during the Dutch East India Company’s colonial administration in Taiwan between 1624 and 1662, recorded in the diaries of the people who maintained its colonial presence (which in Taiwan generally numbered between seven hundred and one thousand). For example, in late 1645, high fever and other diseases spread across Taiwan and resulted in the deaths of over 140 of the Company’s personnel. The deceased included a senior merchant named Stont, and the Dutch hospital was reportedly filled with patients (Cheng, 2000, p.278).

The Qing dynasty’s rule over Taiwan, which lasted approximately 212 years, produced the most extensive documentation on epidemic diseases, primarily consisting of gazetteers from prefectural and county offices, official reports, personal diaries, and travel accounts. For this reason, historical records of epidemics in early Taiwan are predominantly concentrated in the Qing period. During the Qing dynasty, Taiwan (much like mainland China) experienced frequent outbreaks of epidemic diseases. In the 20th year of Emperor Kangxi’s reign (1681), a severe plague afflicted the southern region of the island (Yu, 1984, p.655), and in July of the 60th year of the Kangxi reign (1721) an epidemic ravaged central Taiwan. At the time, Qing troops suppressing the Zhu Yigui rebellion “suffered from acclimatization, resulting in many falling ill and dying.” Additionally, “following the devastation caused by windstorms, the continuous outbreak of epidemics left the people extremely haggard and worn out” (Ding, 1984, p.54-55). These were significant epidemic events during the Kangxi period.

As Taiwan developed, regional outbreaks of disease continued to emerge, reflecting the island’s persistent public health challenges. For instance, Miaoli County experienced an “autumn epidemic” in the 25th year of the Jiaqing reign (1820); a “large-scale epidemic” in the fourth month of summer in the fifth year of the Tongzhi reign (1866); another “autumn epidemic” in the 13th year of the Tongzhi reign (1874); and a “large-scale epidemic” in October of the 15th year of the Guangxu reign (1889) (Shen, 1984, p.128-130). In the Danshui region, located in northern Taiwan, records indicate “a great drought in the summer and an epidemic in the autumn” in the 25th year of the Jiaqing reign (1820). Similarly, in April of the fifth year of the Tongzhi reign (1866), another “large-scale epidemic” occurred (Chen, P., 1984, p.350). Even in the remote Penghu Islands, epidemics were recurrent. Notably, in the 12th year of the Tongzhi reign (1873), “during the winter, the people suffered from a strange epidemic… showing symptoms of a peculiar illness” (Lin, 1984, p.374-375).

In sum, during the Qing dynasty, Taiwan faced recurring epidemics that affected the northern, central, and southern coastal regions of the island, including the outlying Penghu Islands. These significant outbreaks occurred at relatively short intervals and frequently overlapped in terms of both disease type and geographic distribution. Compared to other coastal regions on the Chinese mainland, Taiwan was particularly vulnerable, suggesting the presence of endemic diseases that posed persistent threats to public health.

## Causes and characteristics of frequent epidemics in Qing Taiwan

Previous research suggests that harsh climatic conditions, frequent natural disasters, famines, and wars contributed to epidemic outbreaks in Qing dynasty Taiwan. However, these were primarily aggravating factors rather than fundamental causes; the underlying reason for the recurrent epidemics was Taiwan’s unique natural environment and climatic conditions.

Geographically, Taiwan is an island situated between the tropical and subtropical zones. Taiwan is bisected by the Tropic of Cancer, with the northern half located in the warm subtropical zone and the southern half in the warm tropical zone. However, as a mountainous island situated between the Asian continent and the ocean, Taiwan’s climate is not solely determined by latitude. In fact, the climate of northern Taiwan differs significantly from that of mainland regions at similar latitudes, such as the Minnan-Pearl River region and central Yunnan Province in mainland China. Similarly, southern Taiwan exhibits notable differences from the warm tropical climates of places like the Leizhou Peninsula in Guangdong Province and Hainan Island. Taiwan is also characterized by its mountainous and hilly terrain, with high mountains surpassing one thousand meters accounting for 31% of the island’s total area (Wu, 1979, p.23). This also means that many of the island’s mountainous regions encompass a wide range of climatic zones: from subtropical at the base to subarctic or even alpine conditions at the peaks.

The western part consists mainly of plains and rolling hills, whereas the central and eastern regions are characterized by parallel mountain ranges, many exceeding three thousand meters in elevation. The climate is marked by “high temperatures, abundant rainfall, and strong winds” (Shi, 1987, p.2-3). In winter, prevailing northeast monsoon winds bring strong gusts, especially to the northeast, where overcast and rainy conditions prevail. Summers are long and intensely hot, while spring and autumn are relatively short, a scenario that not only causes considerable physical discomfort but also creates an environment highly favorable to the proliferation of microorganisms and the spread of disease. Warm, humid conditions accelerate bacterial growth, and mosquitoes and other vectors thrive in the island’s vast undeveloped wilderness, especially in forests and swamps, exacerbating health risks. During the early and mid-Qing periods, Taiwan’s dense forests and expansive uncultivated landscapes left newly arrived immigrants highly susceptible to infections, resulting in widespread epidemics. But as Han settlers progressively cleared and cultivated certain hills and plains, epidemic prevalence in those regions declined, and as a result outbreaks in Qing Taiwan often displayed distinct regional patterns, with certain areas exhibiting higher incidence rates.

In the 36th year of the Kangxi reign (1697), Yu Yonghe, a gentry-scholar from Zhejiang, embarked on an expedition to northern Taiwan’s sulfur mines. His journey from south to north offers a vivid portrayal of the environmental and climatic challenges that contributed to disease outbreaks in early Qing Taiwan. Over the course of twenty days, Yu and his companions endured relentless heat, traveling continuously for four days and nights across 96 streams and rivers of varying sizes. The rugged terrain – deep ravines, steep slopes, and sheer cliffs – made their journey arduous. In the lowland plains they encountered dense grasslands, some reaching head height, making movement akin to traveling underground. Swarming insects inflicted constant bites, while the oppressive sun caused their skin to feel as though it might split from the heat, leaving them utterly exhausted (Yu, 1987, p.26). At that time, much of Taiwan remained in a largely untamed and desolate state, teeming with mosquitoes, flies, and venomous insects. The unforgiving natural environment posed severe health risks to immigrants from Fujian and Guangdong, who sought to cultivate the land. During Yu Yonghe’s journey, both he and his entourage succumbed to epidemic illnesses. Among the afflicted were his servants and cook, leaving the group without anyone to prepare food. Reflecting on these hardships, Yu attributed the outbreaks to Taiwan’s remote and inhospitable terrain, where dense vegetation and sparse human habitation allowed miasma to accumulate, leading to widespread disease. He concluded that, given these conditions, it was unsurprising that so many travelers and settlers fell ill (Yu, 1987, p.26).

Yu’s observations suggest that during the early Qing period, epidemic diseases in Taiwan were predominantly concentrated in remote, undeveloped forests and wilderness areas, with “miasma” being the most commonly cited cause. Epidemic outbreaks in Qing Taiwan accordingly exhibited distinct regional characteristics, with Fujianese and Cantonese immigrants particularly vulnerable upon their arrival in these regions. Despite the gradual development of Taiwan in the mid-to-late Qing period, epidemic outbreaks persisted; northern areas such as Danshui and Keelung, as well as southern regions including Taiwan County and Fengshan County, remained endemic foci of disease. In 1772 (the 37th year of the Qianlong reign), Zhu Jingying, an Assistant Commander in Taiwan’s coastal defense forces, described the situation in northern Danshui: “Due to official duties, I arrived at Bali Village and stayed in the southern part of the port. I saw that the mountain tops across the port were shrouded in toxic mist, with an unbearable odor. Previously, patrol offices were established here to guard against this, but due to the noxious air, they were all recently relocated” (Zhu, 1987, p.7-8). An analysis of historical records indicates that certain regions in Taiwan were so inhospitable that even after administrative institutions were established, they were often forced to withdraw due to adverse environmental conditions.

During the late Qing period, Taiwan’s central and eastern regions (along with its southern mountainous areas) remained largely undeveloped and sparsely populated. These regions continued to experience high incidences of epidemic disease. In 1875 (the 1st year of the Guangxu reign), a government report on central and northern Taiwan’s mountainous areas stated: “In locales such as Citongjiao and Suao, miasmatical epidemics are prevalent. Government officials and military personnel deployed to these areas frequently succumb to illness, with Citongjiao reporting particularly high mortality rates” (Erobt, 1984a, p.78). This account underscores the persistence of epidemic disease in Taiwan’s remote mountainous regions during the late Qing period. Qing administrators believed that increasing population density and expanding land development would mitigate miasma-associated illnesses. However, Taiwan’s challenging climate and geography rendered large-scale transformation of its interior particularly difficult.

Ultimately, the prevalence of epidemic disease in Qing Taiwan was intrinsically linked to environmental factors, particularly undeveloped forested regions and uncultivated wilderness. Contemporary sources frequently attributed outbreaks to “miasma” and “vapors,” reflecting the prevailing medical theories of the era. As immigration from Fujian and Guangdong intensified, land development and population growth contributed to a gradual decline in disease incidence. However, Taiwan’s diverse landscape, particularly its mountainous regions, remained resistant to human intervention, ensuring that epidemic outbreaks retained their distinct localized patterns throughout the Qing period.

## Types of epidemic diseases in Taiwan during the Qing dynasty

Historical records from Qing dynasty Taiwan frequently mention epidemic diseases referred to as *yanzhang* [miasma], *zhangqi* [miasmatic vapor], *zhangli/*瘴疠, and *zhangyi/*瘴疫. In traditional Chinese medicine, *zhangqi* is also known as *zhangdu* [miasmatical pestilence] or *zhangli* [miasmatical epidemic], denoting a type of febrile illness caused by exposure to the hot and humid miasmatical vapors of the southern mountainous forests (Ma, 1997, p.417). In ancient times, when medicine was underdeveloped, the understanding of diseases was largely confined to traditional Chinese medical theories and clinical observation. The febrile illness known as *zhangli*, which was attributed to the accumulation of heat and humidity, challenges modern interpretations and remains a complex topic. Even during seventeenth-century Dutch colonial rule, specific causes of the sporadic epidemics could not be determined. At the end of June 1655, a severe epidemic broke out in the Soulang community in southern Taiwan. According to observations by a Dutch official named Cornelis, four to five hundred people were bedridden. The *Zeelandia Diary* recorded the outbreak, stating “This disease has persisted for many years” and “it has deeply taken root, continuously breaking out in various parts of the island, claiming the lives of many indigenous people” (Jiang, 2003, p.499). So even in the mid-seventeenth century, although the Dutch East India Company had established hospitals in Taiwan, the medical methods available to the Dutch at the time were powerless against the epidemics that plagued the island.

What exactly was the *zhangli* epidemic frequently recorded in Qing dynasty Taiwan? Given Taiwan’s geographic environment and climatic conditions, and considering descriptions of its associated symptoms in historical literature, it can be reasonably inferred that *zhangli* likely encompassed a range of severe infectious diseases including malaria, dysentery, cholera, scarlet fever, diphtheria and typhoid fever. However, due to the lack of modern diagnostic methods, the precise types of epidemics involved remain unclear.

In Qing dynasty Taiwan, the most common *zhangli* [miasmatical epidemic] was frequently attributed to exposure to *zhangqi* [miasmatical vapor] or *yanzhang* [miasma]. However, given Taiwan’s rugged mountainous forests and humid climate, it is highly likely that the diseases attributed to *zhang* were, in fact, malaria. The *Gazette of Zhuluo County* from the Kangxi era offers a vivid account of *zhangli*, “In southern Beitou, the miasma induces chills and fever, often leading to delirium. When treated properly and followed by careful convalescence, the disease is easily cured. In northern Beitou, the miasma causes people to become emaciated, with sallow skin, leading to spleen deficiency and symptoms of bloating and distension” (Zhou, 1984, p.292-293). Considering that both “chills and fever” and “bloating and distension” could be the hallmark symptoms of malaria, it is reasonable to assume that many of the so-called “miasmic regions” were likely beset by malaria outbreaks. During this period, the slow development of inland Taiwan with its desolate mountain forests created ideal conditions for mosquitoes to breed, facilitating the spread of the disease. In the mid-Qing dynasty, Zheng Yongxi, a *juren* [provincial graduate] from Hsinchu, composed poems that vividly capture the misery of malarial onset: “When the cold arises, it feels like falling into an icy mountain gorge, the whole body shivering with fear, limbs trembling – even clothed in fox or badger fur, it still feels as thin as gauze; suddenly, the heat comes, scorching like a blazing fire, eyes flickering like stars” (Zheng, 1987, p.9).

In the late Qing dynasty, the Canadian missionary George Leslie Mackay arrived in Taiwan to evangelize and provide medical care. He vividly documented the widespread prevalence of malaria, remarking, “The most harmful and widespread disease that everyone fears is malaria.” Mackay (1972, p.314) further noted that “it is not uncommon to find half the population of a township in Taiwan suddenly stricken with malaria. I have visited villages of twenty or thirty households where not a single person was unaffected.” From this, we can infer that *zhangli*, the predominant epidemic disease in Qing Taiwan, largely referred to malaria. Certainly, traditional medicine during the Qing dynasty lacked the precision of modern diagnostic standards, making it challenging to accurately distinguish and diagnose epidemic diseases in Taiwan. Consequently, historical records describing the symptoms associated with *zhang* (a term encompassing various infectious diseases) suggest that the condition was not limited to malaria but also included other infectious diseases.

Despite the prevailing belief that epidemic diseases in Qing dynasty Taiwan were uniformly related to “miasma” or “noxious vapors” (collectively termed *zhangli*), these outbreaks were in fact diverse and often complex. The terms “miasma” and “noxious vapors” symbolized Taiwan’s harsh and primitive natural environment at the time. In such conditions, the emerging infectious diseases were inevitably varied and frequently compounded, posing greater health risks than typical epidemics and presenting significant challenges to treatment. As Zhu Jingying astutely observed, “those residing in Taiwan, even with minor colds, if administered diaphoretic medicines, may experience weakened internal organs. Should local healers hastily prescribe warming and tonic medicines, unexpected complications can arise. Generally, among those who come here, eight or nine out of ten strong individuals become enfeebled” (Zhu, 1987, p.27). Because traditional Chinese medicine defines disease differently from modern medicine, its approaches to treatment and medication also differ significantly from those used today. As a result, identifying consistent methods of prevention or treatment across different types of epidemics was not something traditional Chinese medicine was particularly adept at, and for this reason epidemic diseases in Qing dynasty Taiwan were often not singular but rather multifaceted, complicating treatment efforts. Moreover, these diseases posed a heightened threat to individuals with compromised health or frequent illnesses. The compound nature of these epidemics, exacerbated by the harsh environmental conditions, resulted in significant health hazards and impeded recovery for many.

## Prevention of epidemic diseases in Qing-ruled Taiwan

The intersection of indigenous practices, immigrant traditions, and environmental conditions shaped Taiwan’s early approaches to epidemic control. During the pre-colonial and colonial periods, Taiwan’s unique position as a migration destination for Han Chinese settlers, combined with its tropical environment, created distinct challenges in disease management and prevention. In 1628, Dutch colonists documented the practice of *Dabung* among seven tribes near Tayouan in southern Taiwan; this ritualistic treatment involved controlled strangulation of seriously ill patients (Blussé, Everts, Frech, 2010, p.81). Female shamans served as primary spiritual healers in these indigenous communities (p.84). This spiritual approach to disease management was mirrored in Han Chinese immigrant communities, where epidemic outbreaks were attributed to supernatural forces. The *Gazette of Zhuluo County* describes widespread shamanistic practices and the elaborate *Wangjiao* ceremony, performed triennially to banish plague deities (Zhou, 1984, p.147-150).

The environmental challenges faced by migrants significantly influenced folk medical practices. Taiwan’s tropical climate, characterized by high humidity and abundant rainfall, shaped both indigenous and immigrant approaches to disease prevention. Han Chinese settlers adapted their medical practices to address what they perceived as environmental health threats, particularly “miasma” and “pestilence.” The widespread consumption of betel nut, mixed with oyster shell powder, emerged as a cultural adaptation to perceived environmental hazards (Chen, W., 1984, p.98). This practice became so deeply embedded that by the late Qing dynasty it transcended gender and age boundaries, with some individuals consuming extraordinary quantities daily (Zhu, 1984, p.71). The environmental consciousness of early Taiwanese society is further evidenced in their water management practices. Recognition of water-borne diseases led to sophisticated purification methods, including the use of alum for water treatment (Erobt, 1984b, p.972). This understanding of environmental risk factors extended to dietary recommendations, with the *Gazette of Zhuluo County* providing specific guidance based on local environmental conditions. The text explicitly connects Taiwan’s damp climate to health risks, advising against excessive pork consumption due to the prevalence of “diseases related to dampness” (Zhou, 1984, p.293).

The evolution of folk medicine in Taiwan represents a synthesis of migrant knowledge and environmental adaptation. Of course, treatment at the time primarily relied on traditional Chinese medicine, a therapeutic system developed over millennia of social practice through which the Chinese discovered that certain plants and animals in nature could alleviate illness. Over time, this knowledge was refined into a systematic medical tradition. It typically identifies the causes of illness based on the patient’s constitution, symptoms and overall condition, and treats disease using combinations of herbs, minerals and animal-derived ingredients, sometimes supplemented by techniques such as acupuncture and stimulation of pressure points. The incorporation of various herbs and treatments demonstrates how settler communities adapted their medical practices to local conditions. In Qing-era Taiwan, popular methods of preventing “miasma” or “pestilential vapors” (*zhangqi*, *zhangli*) included “applying realgar daily to the nostrils and chewing betel nut, in order to fortify one’s courage and spirit so that evil influences could not penetrate” (Erobt, 1984b, p.972). The traditional Chinese remedies for treating epidemic diseases – such as rhinoceros horn, acorus, honeysuckle, curcuma, rehmannia root, forsythia, golden juice (a decoction), belamcanda and burdock, as well as combinations like scrophularia root with honeysuckle dew, golden juice and melon rind – were also widely used in Taiwan during outbreaks of epidemic disease. These treatments were believed to have certain degrees of effectiveness. However, the use of these traditional Chinese methods to prevent epidemics in Qing-era Taiwan faced numerous difficulties. First, importing medicinal materials into Taiwan was not easy and skilled physicians were hard to come by, both common problems of the time. Furthermore, traditional Chinese medicine lacked the precise definitions, diagnostic clarity, and preventive strategies for epidemic diseases that we have today. As a result, treatments were often inconsistent in their effectiveness: sometimes helpful, but often ineffective. This synthesis of indigenous practices, immigrant knowledge and environmental adaptation ultimately contributed to the development of more effective epidemic control measures.

The case of Zhang Zhengduan in Danshui, who successfully treated numerous epidemic victims (Chen, P., 1984, p.451), exemplifies how local medical practices evolved to address Taiwan’s unique epidemiological challenges. This evolution suggests that the intersection of migration patterns, environmental conditions, and medical practices played a crucial role in shaping Taiwan’s early approaches to epidemic management. Moreover, the hybrid nature of medical practices in Taiwan, combining indigenous, immigrant, and colonial knowledge, reflects the broader theme of medical pluralism observed in other colonial contexts, such as South America. This synthesis of medical traditions highlights the adaptability of local communities in the face of environmental and epidemiological challenges.

## The impact of epidemic disease on the development of inland areas in modern Taiwan

The environmental transformation of Taiwan during the late Qianlong period marked a critical juncture in the island’s epidemiological history. By this time, extensive cultivation of the western, northern, and southern plains had significantly altered the natural landscape. However, this anthropogenic environmental change created an uneven development pattern: while coastal regions saw extensive modification, the mountainous interior remained largely untouched, creating distinct disease ecologies that would later impact military operations and colonial ambitions. This ecological contrast lies in the fact that the western plains of Taiwan underwent significant transformation: forests were cleared, wastelands were converted into fertile farmland, and the establishment of villages and towns altered the original natural wilderness. In contrast, the hilly and mountainous regions of central and eastern Taiwan largely remained covered in dense forests; they were remote, sparsely inhabited, and teeming with mosquitoes, flies and venomous insects.

The post-Opium War period introduced new environmental pressures through increased maritime traffic. The opening of treaty ports intensified human movement across the Taiwan Strait, creating new pathways for disease transmission. This environmental-commercial nexus became particularly significant as Western powers began showing greater interest in Taiwan’s strategic position. New ports stimulated Taiwan’s foreign trade, particularly boosting exports of locally produced cash crops such as tea, sugar, and camphor. Notably, both tea cultivation and camphor distillation required workers to venture deep into hilly and mountainous areas, making development of Taiwan’s interior regions significantly more valuable. The resulting changes in human settlement patterns and land use directly influenced the distribution and intensity of epidemic diseases. The correlation between environmental conditions and military effectiveness became starkly apparent during the Japanese invasion of 1874, when the Mudan incident revealed how Taiwan’s tropical environment served as a natural defense mechanism. Japanese casualties overwhelmingly resulted from disease rather than combat: of a total of 4,500 soldiers, 550 died from illness compared to only 12 in battle (Luo, 1987, p.86), showing how environmental factors and particularly endemic diseases shaped military outcomes.

Shen Baozhen’s “Overall Planning for Taiwan” policy represents an early recognition of the link between environmental management and territorial defense. His emphasis on the *Kaishan Fufan* policy (Erobt, 1984a, p.73-76) was essentially an attempt to modify Taiwan’s interior environment to serve strategic needs. The process of opening the mountains required fortresses to be established along mountain routes, troops to be stationed, and settlers recruited to accelerate development of the interior. Naturally, the Qing military’s primary role in *Kaishan Fufan* was to “pacify” what were considered “peaceful tribes” and subdue the “hostile tribes” to ultimately achieve ethnic integration between Han Chinese and the indigenous peoples of Taiwan’s mountainous areas, thereby strengthening Qing rule over the interior. This environmental intervention strategy faced significant biological resistance, however. Deploying 22 battalions along three development routes involved devastating epidemiological challenges, with some battalions reduced to just 27 healthy soldiers (Luo, 1987, p.55). In just over a year of the *Kaishan Fufan* campaign, the impact of epidemic diseases was so severe that nearly three thousand Qing soldiers died from illness, a staggering figure.

By the First Sino-Japanese War (1894-1895), the pattern of environmental influence on military operations reached its apex. Naturally, this also led Japan, during its subsequent colonial rule, to promote the development of regional public health initiatives, improve living conditions in towns and villages, and strengthen the establishment of a modern medical system, all in an effort to change the prevailing conditions that fostered widespread infectious diseases in Taiwan. Despite Japan’s superior military technology, environmental factors still extracted a heavy toll: 26,094 Japanese soldiers fell ill, with 4,642 fatalities from various diseases including malaria, cholera, typhoid, and beriberi. In Penghu alone, 1,247 soldiers succumbed to cholera, highlighting the continued effects of local environmental conditions on military outcomes. The failed attempts to develop Taiwan’s interior regions reveal a crucial insight: the Qing administration’s inability to effectively modify and manage Taiwan’s environment ultimately undermined both public health and defensive capabilities. While epidemic diseases did inflict casualties on foreign invaders, they also prevented the establishment of robust defensive infrastructure and stable settlements in the interior. This environmental-military dynamic demonstrates how ecological factors decisively shaped Taiwan’s late Qing history, ultimately contributing to the Qing dynasty’s failure to establish effective control over Taiwan’s interior and leaving the island vulnerable to Japanese colonial ambitions. The complex interplay between environmental modification, disease ecology, and military effectiveness suggests that successful territorial control required not just military superiority, but also the ability to adapt to and manage local environmental conditions.

## Parallels with South America

The historical trajectories of Taiwan during the Qing dynasty and colonial South America reveal striking parallels in how disease environments shaped territorial development and imperial governance. In both regions, the complex interplay between environmental change, epidemic diseases, and human settlement patterns profoundly affected the success of colonial administration and military operations.

The stabilization of Taiwan’s frontier society, much like early colonial settlements in tropical South America, was persistently undermined by recurrent epidemics and environmental challenges. Collins (1986, p.234) has documented how socioeconomic pressures and inadequate government policies in South American colonies exacerbated environmental damage and public health crises, mirroring the situation in Taiwan where poor land management and rapid settlement created conditions conducive to disease proliferation. The emergence of new infectious diseases in both regions highlights similar patterns of ecological degradation and unsustainable frontier development. The case of Panama disease in South America, as analyzed by Marquardt (2001, p.167), provides a compelling parallel to Taiwan’s “miasma” conditions. Both situations exemplify how monoculture expansion, deforestation, and inadequate land use planning fostered environments favorable to pathogen emergence and spread. McMichael’s (2004, p.45) research further reinforces these connections, highlighting how environmental mismanagement – particularly through land clearing and intensified settlement – created favorable conditions for disease proliferation across diverse geographies.

Taiwan’s position as the last province to be developed in Qing China shares remarkable similarities with the frontier regions of South America, where dense forests and challenging natural environments created what scholars term an “ecological frontier.” In both contexts, the subtropical climate (characterized by high temperatures and humidity) created ideal conditions for infectious disease outbreaks. The spectrum of diseases affecting both regions, which includes malaria, dysentery, cholera, and typhoid, demonstrates the common challenges faced by colonial administrators managing public health in tropical environments. The concept of “ecological imperialism” manifested differently in Taiwan than in the South American colonies, yet both regions experienced what MacLeod and Lewis term “pathological friction” in colonial expansion. While European powers in South America attempted to transform local environments to facilitate control, the Qing administration’s campaign to “open the mountains” in Taiwan revealed similar limitations in environmental modification strategies. Both cases demonstrate how disease environments could simultaneously function as defensive assets and administrative liabilities.

The evolution of medical practices in both regions reflects what Peckham describes as the “hybrid nature” of colonial medicine. Taiwan’s synthesis of Han Chinese folk remedies and indigenous practices parallels the medical pluralism observed in South American colonies, where indigenous knowledge, European medicine, and local adaptations created unique therapeutic landscapes. However, Taiwan’s position as a frontier of Chinese civilization (rather than a site of European colonization) produced distinct dynamics in medical knowledge transmission and environmental management. Comparative analysis of Taiwan and South America highlights the universal challenges of managing disease environments in tropical regions, underscoring the importance of environmental adaptation and medical pluralism in colonial and imperial governance.

This comparison enhances our understanding of how disease environments shaped imperial governance across different colonial contexts. The findings suggest that successful territorial control in tropical environments required not only military superiority but also the capacity to manage local disease ecologies, a challenge that both Qing administrators in Taiwan and colonial powers in South America struggled to overcome. However, the Qing dynasty’s efforts in Taiwan were far from ideal; its attempt to achieve administrative control over the island’s interior through its *Kaishan Fufan* policy to assimilate and control indigenous populations ultimately fell short. Harsh environmental conditions and recurrent epidemics in the mountainous regions prevented the Qing Empire from realizing this goal during the final two decades of its rule. Significant changes only came after the Japanese imperial occupation of Taiwan, as the promotion of public health initiatives and modern medicine under colonial rule transformed both the island’s environment and the patterns of epidemic disease. These parallel experiences provide valuable insights into the historical relationship between epidemics, environmental change, and colonial control, while opening new avenues for research into how similar dynamics played out in other frontier regions across the globe.
